# Gustave Roussy Immune Score Validation in Patients Treated with Bispecific CD3 T-cell Engagers in Phase I Clinical Trials

**DOI:** 10.1158/2767-9764.CRC-26-0171

**Published:** 2026-10-07

**Authors:** Noé Herbel, Clara Helal, Kaïssa Ouali, François-Xavier Danlos, Vincent Goldschmidt, Jean-Marie Michot, Ariane Laparra, Arthur Geraud, Patricia Martin-Romano, Périne Vuagnat, Alice Bernard-Tessier, Anas Gazzah, Rastislav Bahleda, Antoine Hollebecque, Linda Mahjoubi, Clémence Henon, Sophie Postel-Vinay, Yohann Loriot, Vincent Ribrag, Karim Fizazi, Santiago Ponce-Aix, Aurélien Marabelle, Christophe Massard, Stéphane Champiat, Capucine Baldini

**Affiliations:** 1 Drug Development Department, Institut Gustave Roussy, Villejuif, France.; 2 ERC Chromatin Remodeling, DNA Repair and Epigenetics Laboratory, INSERM Unit U981, Institut Gustave Roussy, Villejuif, France.; 3 Faculty of Medicine, Paris-Sud University XI, Paris-Saclay University, Le Kremlin-Bicêtre, France.; 4 Department of Medicine, Institut Gustave Roussy, Villejuif, France.; 5 Department of Medical Oncology, CRCM, CNRS, INSERM, Institut Paoli-Calmettes, Aix-Marseille Université, Marseille, France.; 6 COMPO Team, Centre de Recherche en Cancérologie de Marseille (CRCM), Inserm U1068, Aix-Marseille University, Marseille, France.; 7 University College of London Cancer Institute, London, United Kingdom.; 8 Molecular Radiotherapy Unit 1030, National Institute of Health and Medical Research (INSERM), Paris, France.; 9 Laboratory of Immunomonitoring in Oncology, Institut Gustave Roussy, Villejuif, France.

## Abstract

The Gustave Roussy Immune (GRIm) score, based on the neutrophil-to-lymphocyte ratio, serum albumin, and lactate dehydrogenase, is a validated prognostic marker in patients receiving immune checkpoint blockers (ICB). We evaluated its prognostic value in patients treated with bispecific CD3 T-cell engagers (TCE) in phase I trials. We retrospectively analyzed prospectively collected data from 150 patients treated with TCEs alone or combined with ICBs across 11 phase I trials at the Drug Development Department, Gustave Roussy (June 2016–February 2025). Overall survival (OS) and progression-free survival (PFS) were estimated using Kaplan–Meier methodology and Cox regression models. Discriminatory performance was assessed using Harrell C-index. Among 150 patients, 127 (85%) had solid tumors, and 23 (15%) had hematologic malignancies; 81% were classified as low-risk GRIm score. Low GRIm score was associated with improved OS [hazard ratio (HR), 0.29; 95% confidence interval (CI), 0.17–0.47; *P* < 0.001; median OS, 17.6 vs. 5 months] and PFS (HR, 0.57; 95% CI, 0.36–0.89; *P* = 0.01; median PFS, 2.8 vs. 1.6 months). In solid tumors, the GRIm score remained prognostic for OS (HR, 0.41; *P* = 0.002) but not for PFS. In hematologic malignancies, it strongly predicted both OS (HR, 0.04; *P* < 0.001) and PFS. Multivariable analyses confirmed the GRIm score as independently prognostic for OS (HR, 0.29; *P* < 0.001) and PFS (HR, 0.60; *P* = 0.035). Similar findings were observed in patients receiving TCE in monotherapy. The GRIm score independently predicts outcomes in patients receiving TCEs in phase I trials and may support patient selection, particularly in hematologic malignancies.

**Significance:**

This study validates the use of the GRIm score as a prognostic tool for patients treated with TCE in early-phase trials. It discriminates between low- and high-risk patients across solid and hematologic malignancies and supports the integration of simple biomarkers into patient selection and risk stratification for bispecific CD3 therapies, improving trial design and remaining feasible in routine clinical practice settings.

## Introduction

Patient selection for phase I clinical trials (CT) in oncology and hematology remains a unique challenge because of the complex risk–benefit assessments of these vulnerable populations with advanced disease and a lack of standard treatment options. Conventional primary endpoints of phase I oncology CT used to be the estimation of safety and tolerability profiles in order to identify the maximum tolerated dose and determine the recommended phase II dose of new drugs or drug combinations (1, 2). This design relied on an existing relationship between dose efficacy and dose toxicity for historical treatments (i.e., chemotherapy). Novel anticancer therapies such as anti–PD-1/PD-L1 immune checkpoint blockers (ICB) challenge these traditional drug development processes, as no clear relationship among dose, efficacy, and toxicity has been demonstrated. Recently, the concept of early-phase CTs (EPCT), which are a combination of the conventional phase I and II CTs, has emerged with a dose optimization part (3). In the era of precision oncology, EPCTs enable a preliminary assessment of the efficacy of the tested drug in a small cohort of patients to facilitate rapid access to innovative molecules in the most cost-effective manner. Indeed, reported objective response rates in phase I CTs have increased to around 20%, reaching up to 30% in biomarker-selected populations (4–6). Some promising efficacy data derived from phase I CTs led to several FDA approvals, such as ceritinib, approved for patients with anaplastic lymphoma kinase–rearranged non–small cell lung cancer, and pembrolizumab, approved for patients with melanoma (7–9). Therefore, EPCT now represents a valuable therapeutic option for patients with metastatic cancers, and the optimal selection of patients becomes critical. However, finding the right biomarker remains a challenge, and only few CTs are investigating predictive biomarkers simultaneously. In order to address this question and to better select the patients who might benefit from phase I CTs, several scores have been developed, such as the Royal Marsden Hospital score (RMH score) and the Gustave Roussy Immune (GRIm) score.

The RMH score is a prognostic score predicting survival in patients included in phase I CTs (10), originally designed for cytotoxic and targeted therapies. Three parameters associated with poor survival are used as follows: lactate dehydrogenase (LDH) level (+1: over upper limit normal), albumin level (+1: <35 g/L), and the number of metastases (+1: >2 metastatic sites). According to this score, two prognostic groups of patients can be identified as follows: low-risk (0–1) and high-risk (2–3) patients.

With the development of immunotherapies, the number of phase I CTs testing ICB increased exponentially (11). Therefore, the RMH score’s accuracy was tested when applied to patients treated with ICB but proved to be imperfect. Indeed, the number of metastases was found to be unrelated to prognosis in this population, whereas other markers were identified as new prognostic factors of overall survival (OS), such as a high neutrophil-to-lymphocyte ratio (NLR). Therefore, a new prognostic score better suited for patients treated with ICB emerged: the GRIm score (12). Two of the three variables are common with RMH: LDH level (+1: over upper limit normal) and albumin level (+1: <35 g/L), but the third parameter (i.e., the number of metastases) was replaced with the NLR (+1: NLR > 6). According to the GRIm score, patients can be distributed into two groups: low-risk (GRIm 0 or 1) and high-risk (GRIm 2–3). Since then, new immunotherapy strategies have emerged, such as bispecific CD3 T-cell engagers (TCE; ref. 13).

TCE therapy is based on antibodies that bind both T cells (through CD3) and a tumor-associated (or specific) antigen, constraining the formation of an immune synapse and allowing the bypass of the T-cell receptor/major histocompatibility complex (MHC; ref. 14) interaction. As many tumor cells lose the expression of MHC, bypassing this interaction allows the reactivation of competent T cells from the tumor microenvironment (TME). Target antigen expression constitutes a prerequisite for TCE activity and is currently assessed during clinical development using IHC and molecular imaging approaches to confirm target availability (15). However, increasing evidence suggests that biomarker strategies for TCE therapies will likely extend beyond target expression alone (16). Although antigen density may influence the magnitude of T-cell redirection and cytotoxic activity, characteristics of the tumor immune microenvironment, including baseline T-cell infiltration, IFNγ-related transcriptional programs, and markers of T-cell functional competence, may modulate the ability of redirected T cells to infiltrate tumors and sustain antitumor responses (17). Several additional biomarkers have been explored in the TCE setting, including baseline biomarkers such as circulating tumor DNA and tumor mutational burden, as well as dynamic biomarkers of immune activation, including soluble CD25 (18–20). However, none of these biomarkers have yet been prospectively validated for routine patient selection.

Whether prognostic determinants established in the ICB setting remain relevant in patients treated with TCEs remains largely unknown. Despite mechanistic differences between ICBs and TCEs, both modalities ultimately rely on the quality and availability of the T-cell pool, adequate systemic immune competence, and a host not overwhelmed by tumor burden. These conditions are indirectly captured by the three components of the GRIm score: NLR, serum albumin, and LDH, providing a biological rationale for extrapolating its prognostic value from the ICB setting to TCE-based therapies.

In this context, we conducted a study to evaluate the prognostic value of the GRIm score when applied to patients treated with TCE in phase I CTs.

## Materials and Methods

### Patients

All patients included in a phase I trial at Gustave Roussy’s Early Drug Development Department between June 7, 2016, and February 2025, testing a TCE alone or in association with ICB for advanced hematologic or metastatic solid cancer who received at least one administration of treatment and had a minimum follow-up of 3 months were included in the cohort. Demographic, clinical, and biological data were prospectively collected for each patient during the screening period.

### Endpoints

Target lesions and disease progression were characterized according to Immune-related Response Evaluation Criteria In Solid Tumors (irRECIST) criteria (21) or scintigraphy for bone metastasis. irRECIST was preferred over standard RECIST 1.1 to account for potential pseudoprogression following T cell–mediated immune activation at the tumor site, a pattern increasingly recognized in TCE-treated patients and reflected in the response assessment methodology adopted in landmark TCE trials in solid tumors, including tebentafusp and tarlatamab (18, 22).

Progression-free survival (PFS) was measured from the time of treatment initiation until the time of radiographic progression or death. OS was measured from the day of inclusion until death or the date of the last follow-up. Patients without documented events were censored on the date of the last follow-up visit.

### Statistical methods

Survival data were calculated using the Kaplan–Meier method and compared using a log-rank test (23). Hazard ratios (HR) were calculated using a Cox regression model in univariate analysis (24). The prognostic power of the GRIm score was analyzed using the Harrell C index (25).

The threshold for statistical significance was set at *P* < 0.05. R version 4.4.1 was used to perform statistical analyses.

The study received ethical approval, as granted by the Institutional Review Board (IRB), under the assigned IRB number 2025-501. All patients provided written informed consent prior to participation. The study was conducted in accordance with the ethical principles of the Declaration of Helsinki.

## Results

### Baseline characteristics of the patients

**Figure 1.**
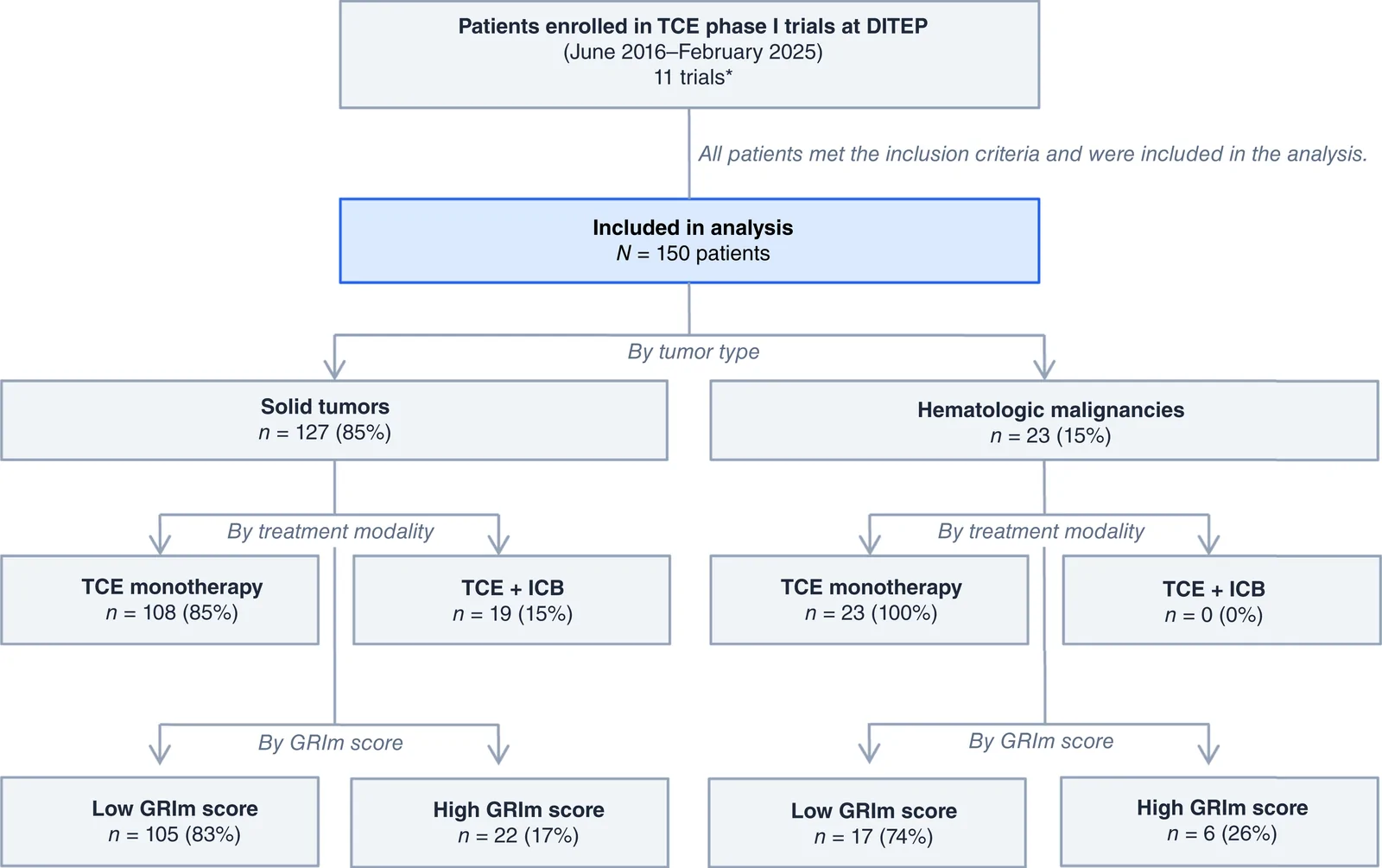
CONSORT flow diagram of participant enrollment and study analysis. *Trial identifiers are confidential and could not be disclosed. DITEP, Drug Development Department.

A total of 150 patients with metastatic solid tumors or advanced hematologic malignancies were enrolled between June 7, 2016, and February 2025 across 11 phase I trials (Tables 1 and 2; Fig. 1). The median age was 61 years (range, 27–82), and 88 patients (59%) were male. At baseline, most patients had normal lymphocyte (64%) and neutrophil counts (88%), and normal albumin levels were observed in 97% of patients. Elevated LDH levels were present in 39% of the cohort. Solid tumors accounted for 85% of cases, with prostate cancer (25%), small cell lung cancer (16%), and B-cell lymphoma (13%) being the most represented tumor types. Among patients with solid tumors, the most common metastatic sites were lymph nodes (69%) and bone (43%). Overall, 81% of patients were classified as low risk according to the GRIm score (Table 1; Fig. 1).

**Table 1.** Baseline characteristics of the patients.

Characteristic (baseline)	Total = 150	
	*N*	%
Gender (male/female)	88/62	59/41
Age (years)	​	​
Median	61	​
Range	27–82	​
ECOG PS	​	​
0	72	48
1–2	78	52
GRIm score	​	​
Low risk	122	81
High risk	28	19
Solid cancer	127	85
Histology	​	​
Prostate cancer	38	25
Small cell lung carcinoma	24	16
High-grade serous ovarian adenocarcinoma	17	11
TNBC	16	11
Colorectal cancer	15	10
Others^a^	17	11
Number of metastatic sites^b^	​	​
1–2	71	56
>2	56	44
Median	2	​
Metastatic sites^c^	​	​
Nodes	87	69
Bones	54	43
Liver	48	38
Lung	39	31
Peritoneum	29	23
Adrenal gland	16	13
Central nervous system	10	8
Hematologic cancer	23	15
Histology	​	​
B-cell lymphoma	20	87
Chronic myeloid leukemia	3	13
Lymphocyte count	​	​
Low (<700/mm^3^)	54	36
PNN count	​	​
ULN (≥7,500/mm^3^)	18	12
NLR	​	​
≥6	42	28
Albumin	​	​
Low (<35 g/L)	4	3
LDH	​	​
ULN (>250 U/L)	59	39
Previous systemic cancer treatment	​	​
Median	3	​
Range	0–9	​
Previous ICBs	30	20

NOTE: The proportion of metastatic sites is calculated among patients with solid cancer.

Abbreviations: PNN, polynuclear neutrophils; ULN, upper limit normal.

a Others: non–small cell lung cancer (3), clear-cell ovarian carcinoma (3), chronic myeloid leukemia (3), glioblastoma (2), cardia adenocarcinoma (2), adenoid cystic carcinoma of the parotid (1), high-grade endometrioid adenocarcinoma (1), pancreatic ductal adenocarcinoma (1), low-grade serous ovarian adenocarcinoma (1), serous adenocarcinoma of the endometrium (1), and uterine carcinosarcoma (1).

b Among patients with solid tumors.

c Only metastatic sites found in at least 10 patients are displayed.

**Table 2.** Baseline characteristics of the patients according to the GRIm score.

Characteristic (baseline)	GRIm low = 122		GRIm high = 28		Fisher exact test	
	*N*	%	*N*	%	OR (95% CI)	*P* value
Gender (male/female)	73/49	60/40	15/13	54/46	​	​
Age (years)	​	​	​	​	​	​
Median	61	​	62	​	​	​
Range	27–82	​	36–79	​	​	​
ECOG PS	​	​	​	​	​	​
0	66	54	6	21	4.28 (1.54–13.83)	0.003
1–2	56	46	22	79	​	​
Solid cancer	105	86	22	79	​	​
Histology^a^	​	​	​	​	​	​
Prostate cancer	30	29	8	36	​	​
Small cell lung carcinoma	18	17	6	27	​	​
High-grade serous ovarian adenocarcinoma	16	15	1	5	​	​
TNBC	14	13	2	9	​	​
Colorectal cancer	13	12	2	9	​	​
Others^b^	14	13	3	14	​	​
Number of metastatic sites^a^	​	​	​	​	​	​
1–2	60	57	11	50	​	​
>2	45	43	11	50	​	​
Median	2	​	2	​	​	​
Metastatic sites^a^	​	​	​	​	​	​
Nodes	72	69	15	68	​	​
Bones	43	41	11	50	​	​
Liver	37	36	11	50	​	​
Lung	30	29	9	41	​	​
Peritoneum	22	21	7	32	​	​
Adrenal gland	14	13	2	9	​	​
Central nervous system	7	7	3	14	​	​
Hematologic cancer	17	14	6	​	​	​
Histology	​	​	​	​	​	​
B-cell lymphoma	14	82	6	100	​	​
Chronic myeloid leukemia	3	18	0	0	​	​
Lymphocyte count	​	​	​	​	​	​
Low (<700/mm^3^)	37	30	17	77	0.28 (0.11–0.72)	0.004
PNN count	​	​	​	​	​	​
ULN (≥7,500/mm^3^)	8	11	10	45	0.12 (0.04–0.42)	<0.001
NLR	​	​	​	​	​	​
≥6	21	17	21	95	0.07 (0.02–0.20)	<0.001
Albumin	​	​	​	​	​	​
Low (<35 g/L)	2	2	2	9	​	​
LDH	​	​	​	​	​	​
ULN (>250 U/L)	37	30	28	100	0 (0–0.06)	<0.001
Previous systemic cancer treatment	​	​	​	​	​	​
Median	2	​	3	​	​	​
Range	0–6	​	1–6	​	​	​
Previous ICBs	22	18	8	36	​	​

NOTE: The proportion of metastatic sites is calculated among patients with solid cancer.

Abbreviations: PNN, polynuclear neutrophils; ULN, upper limit normal.

a Among patients with solid tumors. Fisher test comparisons use the low-risk group as a reference. Nonsignificant comparison results are not displayed.

b Others: GRIm-low: non–small cell lung cancer (3), clear-cell ovarian carcinoma (2), glioblastoma (2), cardia adenocarcinoma (2), adenoid cystic carcinoma of the parotid (1), high-grade endometrioid adenocarcinoma (1), pancreatic ductal adenocarcinoma (2), and low-grade serous ovarian adenocarcinoma (1). GRIm-high: clear-cell ovarian carcinoma (1), serous adenocarcinoma of the endometrium (1), and uterine carcinosarcoma (1).

GRIm score–high patients had a significantly worse performance status, with a higher proportion of Eastern Cooperative Oncology Group performance status (ECOG PS) of 1 to 2 (79% vs. 46%, *P* = 0.003). Lymphopenia was more frequent in these patients (77% vs. 30%, *P* = 0.004), as were elevated neutrophil counts (45% vs. 11%, *P* < 0.001).

Except for albumin, GRIm score features were differentially represented between the two groups. High NLR (95% vs. 17%, *P* < 0.001) and elevated LDH levels (100% vs. 30%, *P* < 0.001) were more frequent in GRIm score–high patients (Table 2).

### OS

**Figure 2.**
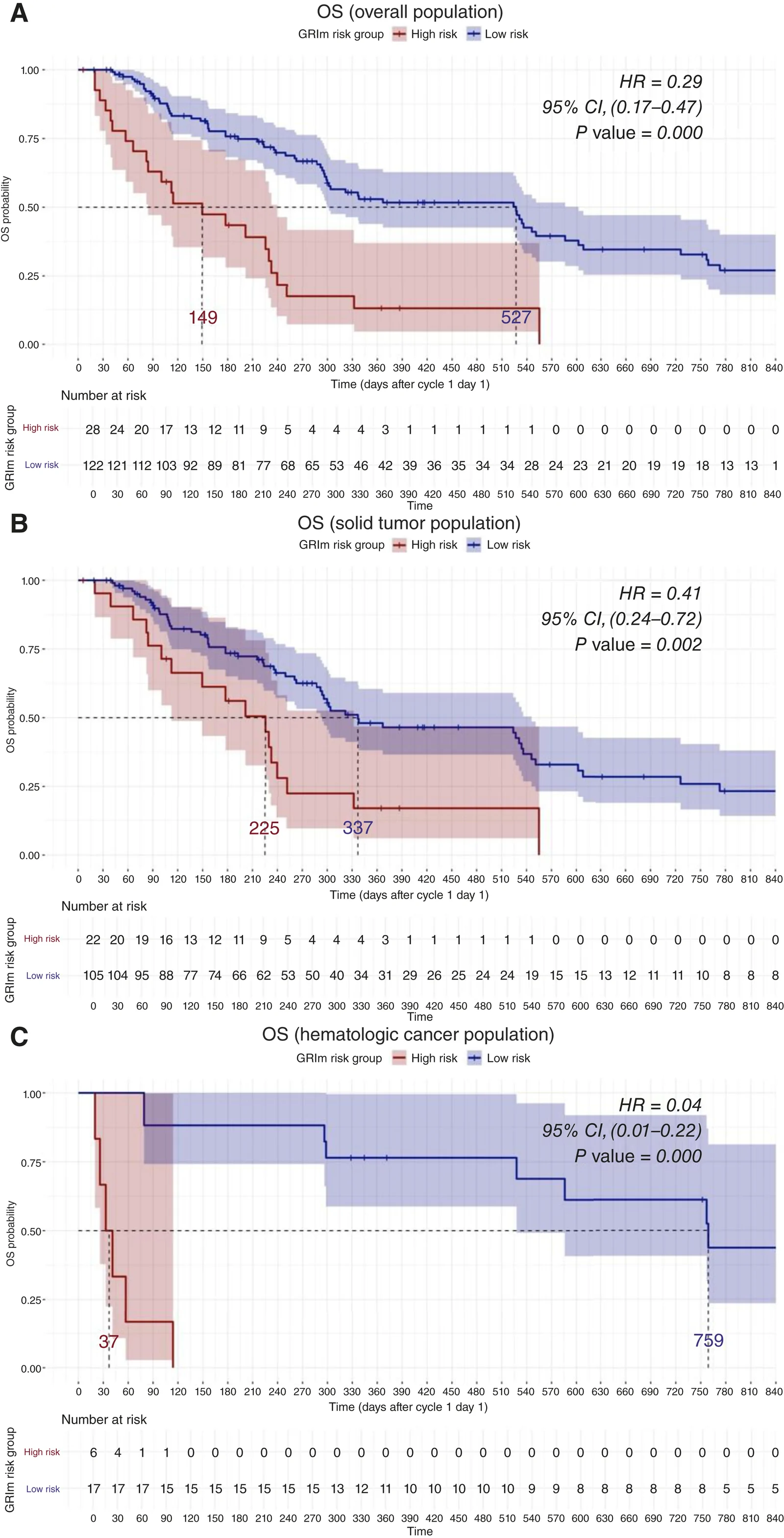
Kaplan–Meier curves for OS according to the GRIm score. OS according to the GRIm score in the overall cohort (**A**) and in patients with solid tumors (**B**) and with hematologic malignancies (**C**). Median OS for each risk group, HR, 95% CI, and *P* value of the comparison of the two GRIm risk groups using Cox regression models are displayed on each plot.

Patients with a low-risk GRIm score at baseline had a better OS compared with a high-risk GRIm score [HR, 0.29; 95% confidence interval (CI), 0.17–0.47; *P* = 2.10–7], with a median OS of 17.6 months (527 days; 95% CI, 300–602) for low-risk patients and 5 months (149 days; 95% CI, 84–239) for high-risk patients (Fig. 2A). Harrell C-index for OS in the overall population is 0.61.

Among patients with a solid tumor, low-risk patients had a better OS compared with high-risk patients (HR, 0.41; 95% CI, 0.24–0.72; *P* = 0.002), with a median OS of 11.2 months (337 days; 95% CI, 294–546) for low-risk patients and 7.5 months (225 days; 95% CI, 112–332) for high-risk patients (Fig. 2B). Harrell C-index for OS in patients with solid tumors is 0.57.

Among patients with a hematologic tumor, low-risk patients had a better OS compared with high-risk patients (HR, 0.04; 95% CI, 0.01–0.22; *P* = 3.10^−7^), with a median OS of 25.5 months (759 days; 95% CI, 528–NA) for low-risk patients and 1.2 months (37 days; 95% CI, 26–NA) for high-risk patients (Fig. 2C). Harrell C-index for OS in patients with hematologic tumors is 0.74.

In Cox multivariate analysis, the GRIm score was associated with OS (HR, 0.29; 95% CI, 0.17–0.5; *P* < 0.001) independently of ECOG PS, age, number of previous treatment lines, combination of treatment (monotherapy or combination with ICB), and cancer type (solid vs. hematologic; Supplementary Fig. S1).

The GRIm score was prognostic of OS for patients treated with monotherapy (HR, 0.27; 95% CI, 0.16–0.46; *P* < 0.001), with a short median OS for high-risk patients (5 months) compared with low-risk patients (17.8 months; Supplementary Fig. S2).

### PFS

**Figure 3.**
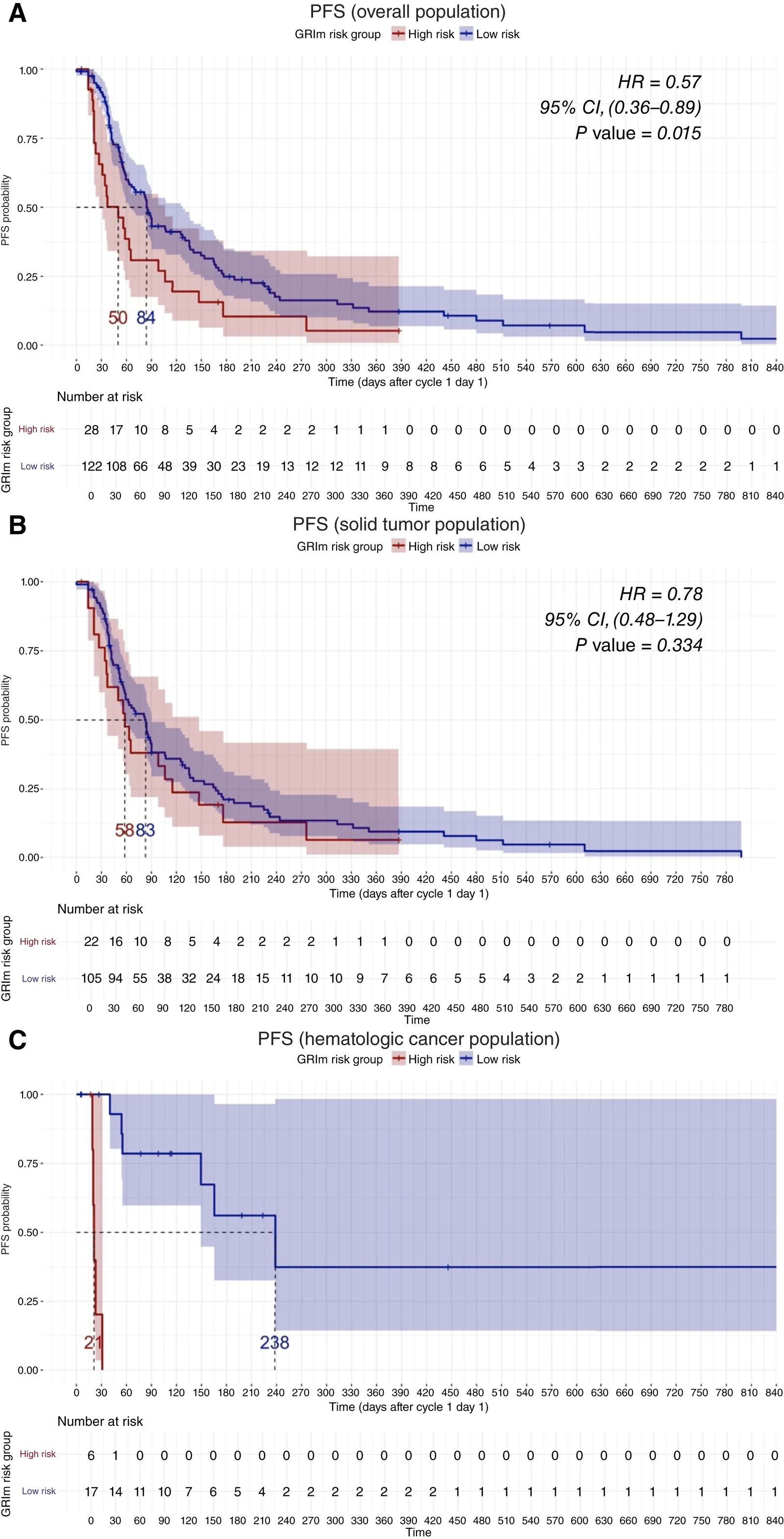
Kaplan–Meier curves for PFS according to the GRIm score. PFS according to the GRIm score in the overall cohort (**A**) and in patients with solid tumors (**B**) and with hematologic malignancies (**C**). Median PFS for each risk group, HR, 95% CI, and *P* value of the comparison of the two GRIm risk groups using Cox regression models are displayed on each plot, except for **C**, in which every patient from the high-risk group had progressive disease before the first in the other group.

The Cox regression model on PFS showed a better outcome for patients in the low-risk group (HR, 0.57; 95% CI, 0.36–0.89; *P* = 0.01; Fig. 3A). The median PFS was 2.8 months (84 days; 95% CI, 63–127) for low-risk patients and 1.6 months (50 days; 95% CI, 27–106) for high-risk patients. The Harrell C-index for PFS in the overall population is 0.56.

Among patients with a solid tumor, no significant difference was observed between high- and low-risk patients for PFS (HR, 0.78; 95% CI, 0.48–1.29; *P* = 0.33; Fig. 3B). The Harrell C-index for PFS in patients with solid tumors is 0.53.

Among patients with a hematologic tumor, all high-risk patients progressed before the first low-risk patient (Fig. 3C). The median PFS was 7.9 months (238 days; 95% CI, 149–NA) for low-risk patients and 0.7 months (21 days; 95% CI, 20–NA) for high-risk patients. The Harrell C-index for OS in patients with hematologic tumors is 0.79.

In Cox multivariate analysis, the GRIm score was associated with PFS (HR, 0.6; 95% CI, 0.37–0.96; *P* = 0.035) independently of ECOG PS, age, number of previous treatment lines, combination of treatment (monotherapy or combination with ICB), and cancer type (solid vs. hematologic; Supplementary Fig. S3).

The GRIm score was prognostic of PFS for patients treated with monotherapy (HR, 0.56; 95% CI, 0.35–0.91; *P* = 0.019), with a short median PFS for high-risk patients (1.7 months) compared with low-risk patients (2.8 months; Supplementary Fig. S4).

## Discussion

Here, we report the retrospective validation of the GRIm score as a useful biomarker for patient selection in phase I CTs evaluating TCE for solid or hematologic malignancies. In phase I CTs, identifying patients who are most likely to benefit from experimental treatments is crucial for selecting the appropriate candidates. Furthermore, stratifying patients based on biomarkers is essential to improve survival and quality of life by optimizing treatment efficacy and minimizing severe adverse events from ineffective treatments.

Our study demonstrates that the GRIm score is associated with the OS of phase I CT patients treated with a TCE for either a solid or hematologic malignancy. To our knowledge, this study is the first to validate a prognostic score for patients treated with TCE. Although the number of patients with hematologic cancer was relatively low, this study also showed that risk groups based on the GRIm score were significantly prognostic of PFS specifically in patients with hematologic malignancies, whereas no significant association with PFS was observed in patients with solid tumors (HR, 0.78; 95% CI, 0.48–1.29; *P* = 0.33). Importantly, the independent prognostic value of the GRIm score was confirmed in a multivariable Cox model adjusting for ECOG performance status, age, number of prior treatment lines, cancer type (solid vs. hematologic), and treatment modality (TCE monotherapy vs. TCE + ICB combination), for both OS (HR, 0.29; 95% CI, 0.17–0.5; *P* < 0.001) and PFS (HR, 0.60; 95% CI, 0.37–0.96; *P* = 0.035), confirming that the prognostic signal of the GRIm score is not attributable to imbalances in these clinical covariates (Supplementary Figs. S1 and S3). Furthermore, to address the potential confounding effect of ICB coadministration on survival outcomes, a subgroup analysis restricted to patients treated with TCE monotherapy demonstrated that the GRIm score retained strong prognostic significance for OS (HR, 0.27; 95% CI, 0.16–0.46; *P* < 0.001; median OS, 17.8 vs. 5 months) and PFS (HR, 0.56; 95% CI, 0.35–0.91; *P* = 0.019; median PFS, 2.8 vs. 1.7 months) in this subgroup (Supplementary Figs. S2 and S4), indicating that the prognostic value of the GRIm score is independent of ICB coadministration and reflects host factors intrinsically relevant to TCE-mediated antitumor activity.

The good discriminatory value of the GRIm score relies on the combination of three well-known parameters associated with poor prognosis: NLR, albumin, and LDH levels.

First, the NLR is an inflammation-related marker, reflecting the balance between the innate immune response (mainly supported by neutrophils) and adaptive immunity (relying on lymphocytes; ref. 26). It was widely demonstrated that a high NLR is an independent prognostic factor associated with poor short- and long-term outcomes in both solid and hematologic malignancies (27). Indeed, neutrophils provide a favorable microenvironment for tumor cell proliferation, whereas the reduction in lymphocyte counts suppresses the antitumor response (poor T-cell cytotoxic activity; ref. 28). Such an inflammatory and immunosuppressive TME is not favorable for TCE’s efficacy.

Then, a low albumin level is a marker linked with the poor nutritional status of patients with cancer and is usually associated with a low ECOG PS. Studies have shown that a low albumin level is an independent prognostic factor associated with poor OS (29). Therefore, this parameter can be used as an exclusion criterion in CTs (30, 31).

Finally, a high plasma LDH level reflects high metabolic activity linked to a high tumor burden in patients with cancer. Studies recently showed that a high plasma LDH level is related to immunosuppression (32) and is significantly associated with shorter PFS and OS in cancer (33).

Other studies validated the GRIm score as a prognostic parameter in different populations of patients treated with various classes of treatments, such as Folinic Acid, Irinotecan, Oxaliplatin, and 5-Fluorouracil combination or gemcitabine and nab-paclitaxel in pancreatic adenocarcinoma, platinum-based chemotherapy in small cell lung cancer, or surgical resection in esophageal squamous cell carcinoma, in patients treated with first-line chemotherapy for metastatic colorectal cancer, in patients with early breast cancer, and in platinum-refractory patients with metastatic urothelial carcinoma treated with pembrolizumab (34–38). Meta-analyses in patients with lung and gastrointestinal cancers have also shown the GRIm score to have prognostic value (39, 40). A pan-cancer meta-analysis showed similar results (41).

In addition, changes in the GRIm score under treatment have also been described as a marker of sensitivity to nivolumab in advanced gastric cancer (42). However, to enhance its prognostic value for a given tumor type, the GRIm score has been modified to better suit the specificity of each malignancy. A modified GRIm score, based on the same parameters but with different cutoffs (albumin, 37.3 g/L; LDH, 193 U/L; and NLR, 2.48), was validated in patients who underwent surgery for proximal gastric cancer (43). The hepatocellular carcinoma (HCC)–modified GRIm score was also validated on patients treated with atezolizumab and bevacizumab for HCC. The HCC-modified GRIm score is based on the same parameters with two new cutoffs (LDH > 245 U/L and NLR > 4.8) and two new parameters: aspartate aminotransferase (AST)–to–alanine aminotransferase (ALT) ratio (AST/ALT > 1.44: +1) and total bilirubin level (>1.3 mg/dL: +1). Based on the HCC-modified GRIm score, patients with up to two points are considered low-risk, whereas patients with a higher score are considered high-risk (44). Consistent with these previous studies, our findings suggest that the GRIm score could serve as a valuable prognostic biomarker for phase I CT patient selection involving TCE treatment. Nevertheless, its prognostic performance may not be uniform across malignancies and treatment contexts. Several biological and methodologic factors may explain the superior discrimination of the GRIm score in hematologic versus solid malignancies. First, hematologic tumors, particularly B-cell lymphomas, are more uniformly accessible to TCE-mediated T-cell killing, with fewer stromal barriers than solid tumors, making systemic immune parameters more directly reflective of prognosis and treatment outcomes. Second, the extensive prior therapies frequently received by patients with relapsed/refractory hematologic malignancies may accentuate differences in systemic immune status captured by GRIm score components. Third, the heterogeneity of solid tumor types and microenvironments in our cohort may dilute the prognostic signal of the GRIm score, which was designed in a broader, tumor-agnostic context. Taken together, these findings raise the possibility that disease-specific refinement of the GRIm score, including distinct calibration for hematologic versus solid malignancies, could further improve its prognostic performance.

Moreover, the prospective study of the French REISAMIC showed that in the context of ICB, the NLR was a predictive factor for developing very severe and fatal adverse events (grade 5), and albumin level was a predictive factor for the fatal adverse event registry (45). Interestingly, two of the three parameters composing the GRIm score have therefore individually been associated with severe toxicity risk in the immunotherapy setting. Even though their mechanisms of action are different, both ICBs and TCEs rely on immune modulation and can induce severe immune-mediated toxicities (cytokine release syndrome, tumor flare, etc.; ref. 46). This raises the hypothesis that baseline systemic inflammatory and nutritional status, as captured by the GRIm score, may also influence toxicity risk in patients receiving TCE therapies. However, whether the GRIm score could serve not only as a prognostic biomarker but also as a predictor of TCE-related immune toxicity, particularly cytokine release syndrome, remains an open question beyond the scope of this prognostic study and warrants prospective evaluation in dedicated cohorts.

The main limitations of our study include the small size of the hematologic cohort (*n* = 23). The extreme HR observed (0.04) should be interpreted with caution and prospectively validated in dedicated, larger cohorts. Another limitation is the higher proportion of patients with a low (0–1) GRIm score (81%). This overrepresentation of low-risk patients likely reflects an inherent selection bias of phase I CTs, whose eligibility criteria systematically exclude patients with poor performance status, severe organ dysfunction, or advanced nutritional impairment, characteristics frequently associated with higher GRIm scores. Consequently, the limited number of high-risk patients included in our cohort (*n* = 29) may have reduced the statistical power to fully characterize this subgroup and may limit the generalizability of our findings to more clinically vulnerable populations. In addition, even though the GRIm score was found to be prognostic of OS in every setting, its performance in solid tumors seems more limited in patients treated with TCEs compared with those treated with ICBs (C-index of 0.7 for OS in ICB and 0.57 in TCE). Better prognostic and predictive tools need to be developed to refine patients’ selection for phase I CTs testing TCEs, either by modifying the GRIm score as previously demonstrated with disease-specific adaptations such as the HCC-modified GRIm score or by building a new score. Future refinements should also consider whether distinct calibration strategies are required for hematologic versus solid malignancies, given their marked biological differences and the differential prognostic performance observed in our cohort. Prospective validation across larger cohorts and multiple tumor types will be required to determine whether disease-specific adaptations or dedicated TCE-oriented prognostic models could further improve patient stratification.

### Conclusion

Overall, our analysis demonstrated that the GRIm score is a valuable tool to better select phase I CT patients for the evaluation of a TCE in solid or hematologic malignancies based on OS. It was also found to be heavily prognostic of PFS in patients with hematologic malignancies. Nevertheless, the GRIm score failed to be prognostic of PFS for patients with solid tumors.

As it is a minimally invasive, low-cost, and objective blood count–based biomarker, we support that the use of the GRIm score should be routinely integrated into the patient selection process for TCE treatment, especially in patients with hematologic malignancies. Still, better prognostic tools need to be developed for the selection of patients with solid tumors.

## Supplementary Material

Figure S1 Multivariate analysis shows the GRIm score is independently prognostic of OS.

Figure S2 Kaplan Meier curves for OS according to the GRIm score in patients treated with TCE in monotherapy.

Figure S3 Multivariate analysis shows the GRIm score is independently prognostic of PFS.

Figure S4 Kaplan Meier curves for PFS according to the GRIm score in patients treated with TCE in monotherapy.

## Data Availability

The clinical data collected and analyzed during this study are not publicly available due to patient confidentiality, privacy considerations, and ethical restrictions. The data cannot be shared because informed consent and institutional approvals do not permit public dissemination of individual-level clinical data.
