# Supplementary material for: Gustave Roussy Immune Score Validation in Patients Treated with Bispecific CD3 T-cell Engagers in Phase I Clinical Trials
Source: Cancer Res Commun. 2026 Oct 7;6(10):2339–49. doi: 10.1158/2767-9764.CRC-26-0171 (PMC13643720; doi:10.1158/2767-9764.CRC-26-0171)
Supplement: Supplementary file 1 [file crc-26-0171_figure_s1_suppsf1.pdf]

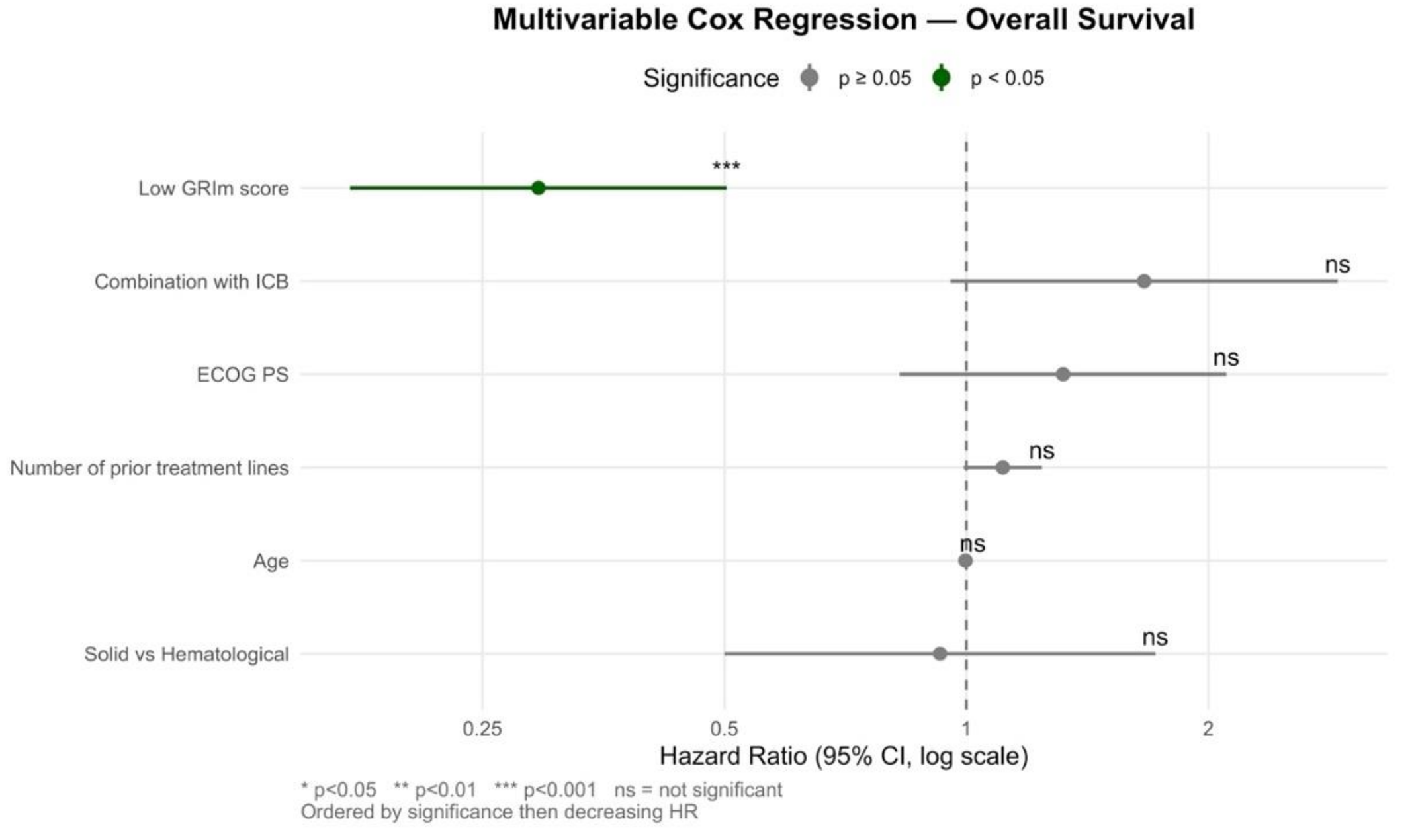

Multivariable Cox Regression — Overall Survival
Significance
p ≥ 0.05
p < 0.05
Low GRIm score
***
Combination with ICB
ns
ECOG PS
ns
Number of prior treatment lines
ns
Age
ns
Solid vs Hematological
ns
0.25
0.5
1
2
Hazard Ratio (95% CI, log scale)
* p<0.05   ** p<0.01   *** p<0.001   ns = not significant
Ordered by significance then decreasing HR

Figure S1 : Multivariate analysis shows the GRIm score is independently prognostic of OS .

Cox multivariate analysis was conducted on all displayed parameters. GRIm score, Combination with ICB, ECOG PS and solid vs hematological were used as categorical variables. Number of prior treatment lines and Age were used as continuous variables.

GRIm : Gustave Roussy Immune score ; ICB : Immune checkpoint blockers ; ECOG PS : Eastern Cooperative Oncology Group performance status – cut off used = 0 vs 1-2.
