# Supplementary material for: Gustave Roussy Immune Score Validation in Patients Treated with Bispecific CD3 T-cell Engagers in Phase I Clinical Trials
Source: Cancer Res Commun. 2026 Oct 7;6(10):2339–49. doi: 10.1158/2767-9764.CRC-26-0171 (PMC13643720; doi:10.1158/2767-9764.CRC-26-0171)
Supplement: Supplementary file 2 [file crc-26-0171_figure_s2_suppsf2.pdf]

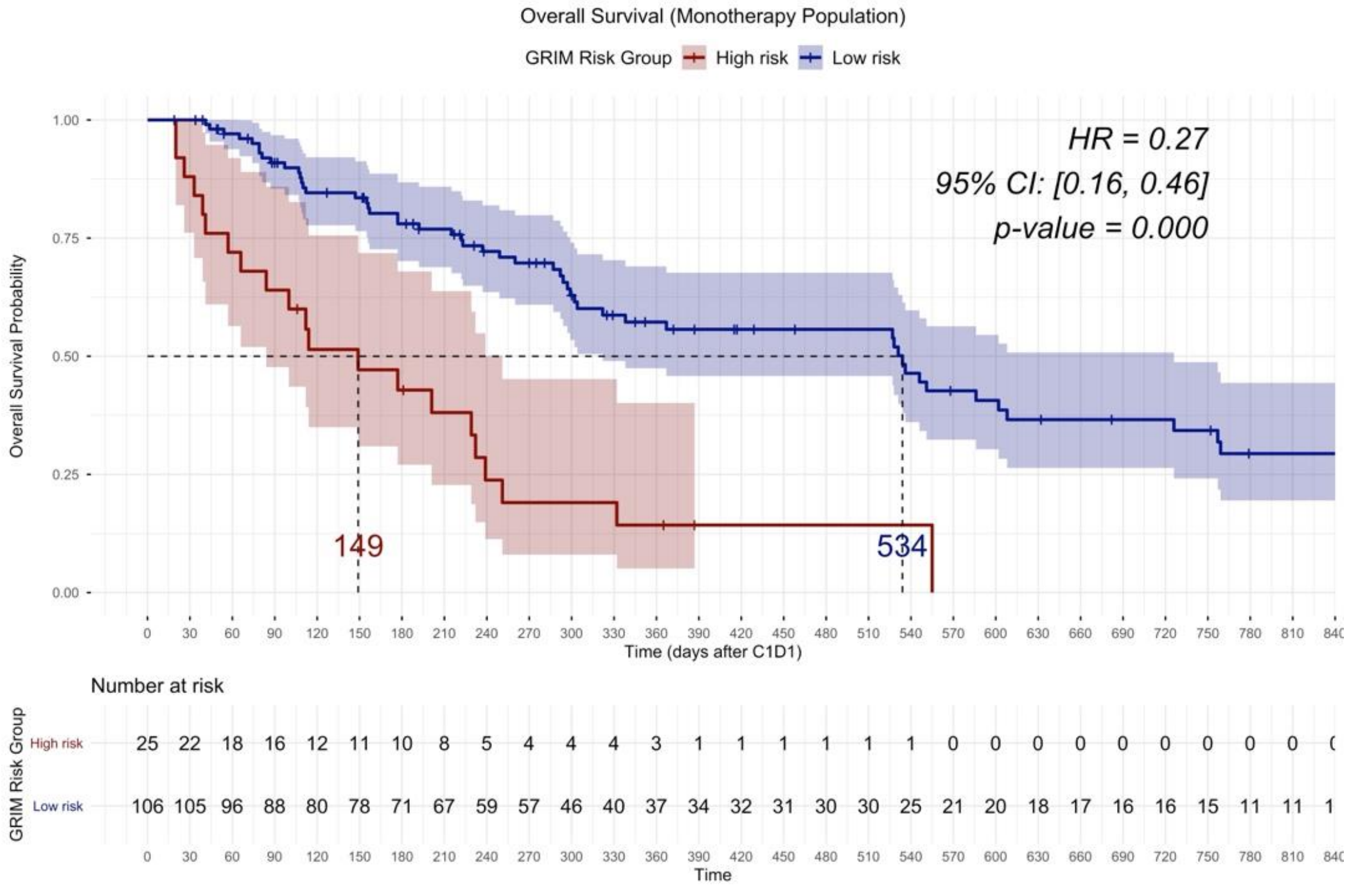

Overall Survival (Monotherapy Population)
GRIM Risk Group
High risk
Low risk
HR = 0.27
95% CI: [0.16, 0.46]
p-value = 0.000
Overall Survival Probability
1.00
0.75
0.50
0.25
0.00
149
534
Time (days after C1D1)
Number at risk
GRIM Risk Group
High risk
25 22 18 16 12 11 10 8 5 4 4 4 3 1 1 1 1 1 1 0 0 0 0 0 0 0 0 0
Low risk
106 105 96 88 80 78 71 67 59 57 46 40 37 34 32 31 30 30 25 21 20 18 17 16 16 15 11 11
0 30 60 90 120 150 180 210 240 270 300 330 360 390 420 450 480 510 540 570 600 630 660 690 720 750 780 810
Time

Figure S2. Kaplan Meier curves for OS according to the GRIm score in patients treated with TCE in monotherapy.
