# Supplementary material for: Gustave Roussy Immune Score Validation in Patients Treated with Bispecific CD3 T-cell Engagers in Phase I Clinical Trials
Source: Cancer Res Commun. 2026 Oct 7;6(10):2339–49. doi: 10.1158/2767-9764.CRC-26-0171 (PMC13643720; doi:10.1158/2767-9764.CRC-26-0171)
Supplement: Supplementary file 3 [file crc-26-0171_figure_s3_suppsf3.pdf]

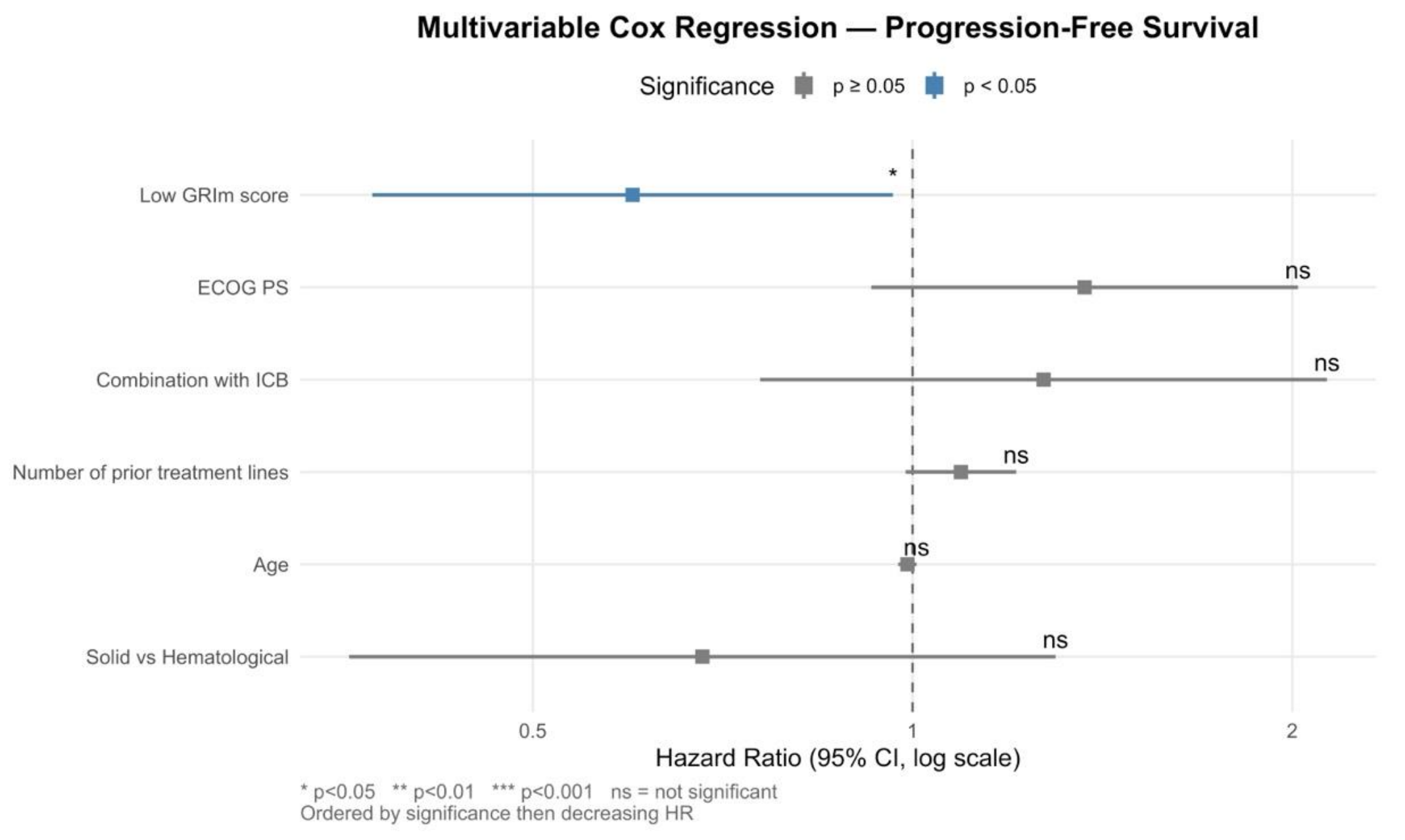

Multivariable Cox Regression — Progression-Free Survival
Significance p ≥ 0.05 p < 0.05
Low GRIm score
ECOG PS
Combination with ICB
Number of prior treatment lines
Age
Solid vs Hematological
*
ns
ns
ns
ns
ns
0.5
1
2
Hazard Ratio (95% CI, log scale)
* p<0.05 ** p<0.01 *** p<0.001 ns = not significant
Ordered by significance then decreasing HR

Figure S3 : Multivariate analysis shows the GRIm score is independently prognostic of PFS.
Cox multivariate analysis was conducted on all displayed parameters. GRIm score, Combination with ICB, ECOG PS and solid vs hematological were used as categorical variables. Number of prior treatment lines and Age were used as continuous variables.
GRIm : Gustave Roussy Immune score ; ICB : Immune checkpoint blockers ; ECOG PS : Eastern Cooperative Oncology Group performance status – cut off used = 0 vs 1-2.
