# Supplementary material for: Gustave Roussy Immune Score Validation in Patients Treated with Bispecific CD3 T-cell Engagers in Phase I Clinical Trials
Source: Cancer Res Commun. 2026 Oct 7;6(10):2339–49. doi: 10.1158/2767-9764.CRC-26-0171 (PMC13643720; doi:10.1158/2767-9764.CRC-26-0171)
Supplement: Supplementary file 4 [file crc-26-0171_figure_s4_suppsf4.pdf]

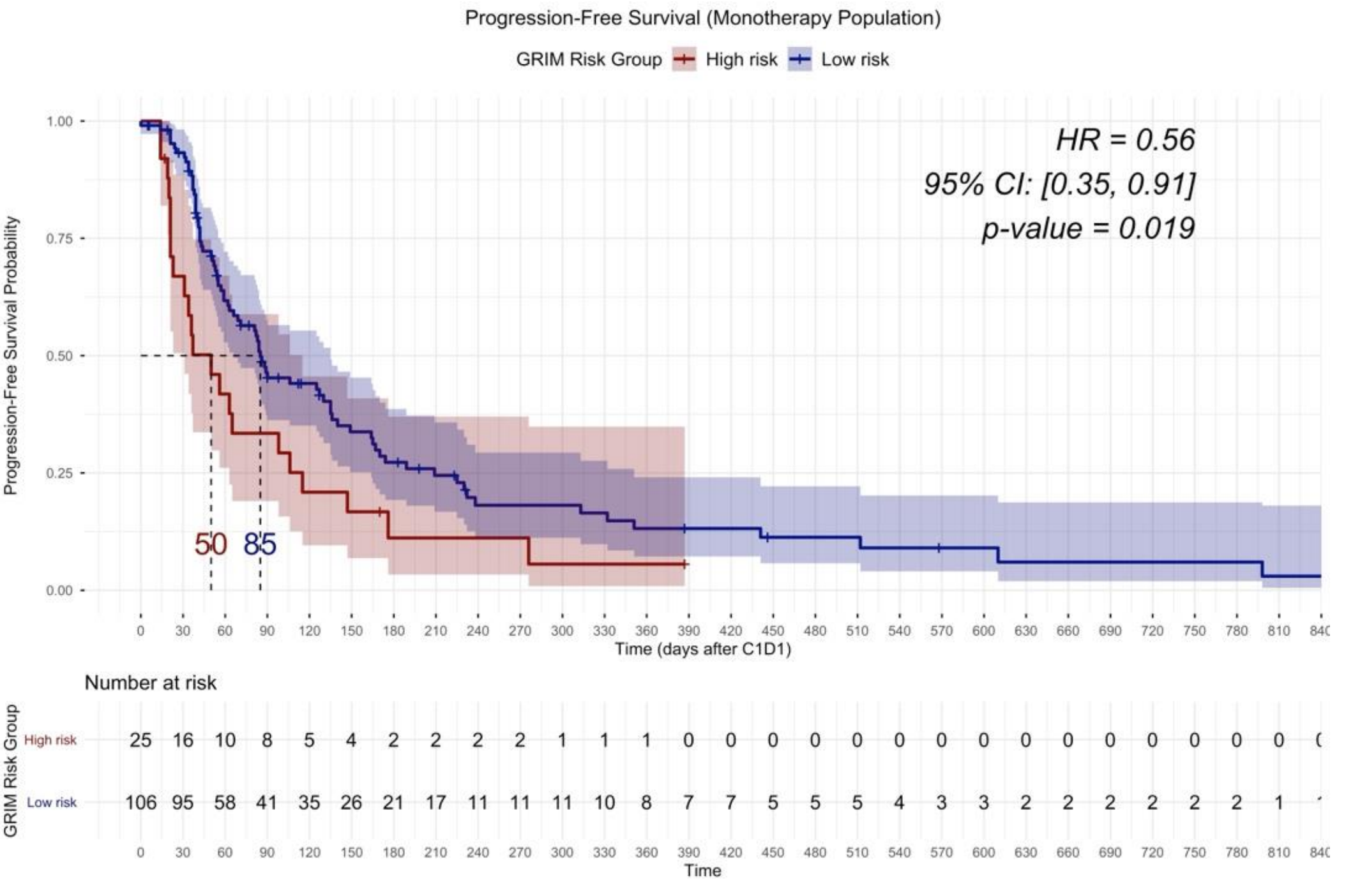

Progression-Free Survival (Monotherapy Population)
GRIM Risk Group
High risk
Low risk
HR = 0.56
95% CI: [0.35, 0.91]
p-value = 0.019
Progression-Free Survival Probability
1.00
0.75
0.50
0.25
0.00
50
85
0 30 60 90 120 150 180 210 240 270 300 330 360 390 420 450 480 510 540 570 600 630 660 690 720 750 780 810 840
Time (days after C1D1)
Number at risk
GRIM Risk Group
High risk 25 16 10 8 5 4 2 2 2 2 1 1 1 0 0 0 0 0 0 0 0 0 0 0 0 0 0 0 0
Low risk 106 95 58 41 35 26 21 17 11 11 11 10 8 7 7 5 5 5 4 3 3 2 2 2 2 2 2 1 1
Time

Figure S4. Kaplan Meier curves for PFS according to the GRIm score in patients treated with TCE in monotherapy .
